# Iodine a Substitute for Quinia

**Published:** 1880-07-20

**Authors:** Fordyce Grinnell

**Affiliations:** Maryville, Tenn.


### IODINE A SUBSTITUTE FOR QUINIA.

BY FORDYCE GRINNELL, M. D., MARYVILLE, TENN.

While occupying the position of Government physician at the Wichita Indian Agency, Indian Territory, I saw a statement quoted from the St. Petersburger Medical Dochenschrift on the value of iodine as a substitute for quinia. The statement made by the author, Dr. J. Nonodnitschauski, that "when given boldly, ten to twelve drops of the tinct. in a half glass of sweetened water every eight hours, iodine will never rank second to quinia in the treatment of intermittent fevers," the more forcibly impressed me, because at the time malarial diseases were prevailing extensively, and while I had been using the sulphate of quinia at the rate of one ounce per day, my stock suddenly became exhausted, and no article ordinarily used as a substitute remained in the dispensary. Our distance from medical supplies rendered our condition, under these circumstances, very embarrassing. Hence, the readiness with which we seized upon any suggestions which seemed to afford means to fight our great enemy—malaria.

Having then a good opportunity to test the value of the remedy, I began by following the plan suggested by Dr. Nonodnitchauski, that is, giving ten drops of the tinct. in one-third glass of sweetened water, thrice daily to adults, children receiving proportional doses. The results far surpassed my most sanguine expectations.

Indeed, I thought the statement rather extravagant, that iodine when given as above indicated, "will never rank second to quinia in the treatment of intermittent fevers."

Subsequent experience however, both in that country and in this, has led me to conclude that the anti-periodic powers of iodine are superior to any other remedy of the materia medica save quinine, and that it is by far the best known substitute for quinia.

At that time I treated 135 cases of intermittent fever, 74 being males and 61 females; these included children and in some instances infants. The quotidian and tertian types of the fever were the forms principally presented. I also treated four cases of diarrhœa and eight cases of neuralgia, each of malarial origin, using the same remedy, only adding astringents or opiates as indicated. One hundred and forty-seven cases were thus treated with the iodine, and the results were fully equal to those treated with the sulphate of quinia.

The remedy seemed to act almost as by magic, in many instances the paroxysms were not repeated after the medicine was given, though the doses were repeated for a day or two after the cessation of the fever.

In cases of enlarged spleen there was a more speedy reduction in the size of that organ than when the sulphate of quinia was used.

One important item in its favor was the fact that it was much more agreeable to take than quinine, and this with a large part of our population proved a potent argument in its favor. The iodine at once became a more popular remedy than quinine, with the masses of our people.

The nationality of those treated embraced the white, the Indian and the negro races.

Knowing that some of my brother practitioners at other agencies were also short of anti-periodic remedies, I at once reported my successes to my friend, Dr. Irving W. Smith, physician to the Kiowa and Comanche Agency. He reported some time after—"I have tried the new remedy in a number of instances, with both red and white patients, in each instance with complete success as far as reported. I have only a few ounces of quinia or cinchonidia, and regard the new remedy as a special blessing at this time. I have added the tinct. to simple syrup, a drachm to the ounce, with enough iodide of potassum to prevent precipitation on the addition of water."

I also reported upon the value of iodine as a substitute for quinia to the Cincinnati Lancet and Clinic, and several succeeding numbers contained testimonials from various parts of our country as to its decided value. Since coming to Tennessee I have had fewer cases of malarious character with which to deal, but in these few I am perfectly satisfied with its results, as they have been fully equal to those recorded in my government practice.

Several practitioners along the Tennessee river, where miasmatic fevers are more prevalent, have, at my seggestion, used the iodine, and they too have been astonished at the favorable results. In one instance the doctor informed me that he had used quinia and the various alkaloids of cinchona without avail in an obstinate case of malarial fever in a child, that he had also tried arsenic and other anti-periodic remedies of repute, but without success; that he finally prescribed the iodine, with a perfect cure as the result. Previous to this he said he had so little faith in the remedy (although it had been proposed to him some time before this) that he had not tried it, and only because of want of success in the use of all other remedies had he tried it in this instance, but he now proposed to give it a further trial.

The fact that the cost of iodine is so little in comparison to quinia, especially renders it a boon to the poorer classes, who can ill afford to purchase quinia, and the physician who treats them and furnishes the remedy, finds his bill for drugs not an insignificant item of his expenses, with little to show in return.

The fact that iodine exerts such a pronounced effect upon those very glands which appear to harbor the malarial poison, seems to render its great value in these cases quite philosophical.

It only seems strange that in the settling up of the great West, with malarial poison ever opposing the onward march of civilization, and the great scarcity and high price of quinia, that the value of this remedy had not been earlier and more generally appreciated.

It is safe to say that hundreds of persons have died of malarial disorders, especially in the territories and regions remote from medical supplies, because of the scarcity of quinia, or because it could not be obtained at any price, when perhaps at the same time iodine in quantity was in stock in the dispensaries.—*South. Prac.*

